# *HLA Class Ib* and *MICA/MICB* Expression in Human Tissues and Cell Types: Reshuffling Immune Players

**DOI:** 10.1111/tan.70390

**Published:** 2025-09-10

**Authors:** Laurent Abi‐Rached, Pierre Faux, Julien Paganini, Jacques Chiaroni, Pierre Pontarotti, Julie Di Cristofaro

**Affiliations:** ^1^ Aix Marseille Univ, MEPHI Marseille France; ^2^ IHU Méditerranée Infection Marseille France; ^3^ CNRS UR D‐258, SNC5039 Marseille France; ^4^ GenPhySE, Université de Toulouse, INRAE, ENVT Castanet Tolosan France; ^5^ Xegen Gemenos France; ^6^ Aix Marseille Univ, CNRS, EFS, ADES Marseille France; ^7^ Etablissement Français du Sang PACA Corse Marseille France

**Keywords:** HLA, MICA/B, physiological expression, RNAseq, single‐cell

## Abstract

Abnormal expression of HLA class Ib, MICA and MICB molecules is associated with the evolution of pathological conditions and clinical settings. Here, we use RNA‐sequencing data from two publicly‐available projects, from different human organs and tissues and at single‐cell level, to present their transcriptional expression throughout the human body, in comparison to that of *HLA class Ia, HLA class II,* their costimulatory molecules, and the main *HLA* transcription factors. Our analyses for 21 target genes reveal that median gene expression differs by orders of magnitude and that the classical/non‐classical HLA distinction is not absolute for overall expression. Sixteen of the 21 target genes show correlated expressions, although careful analyses of individual expression patterns in tissues and organs highlight specificities. Tissue and organ expression patterns reveal that the lymphoid organs, lungs, and gastrointestinal tract organs display the highest expression of the *HLA* and *HLA‐related* genes. At single‐cell level, adipocytes, endothelial cells, and immune cells all have unexpectedly close expression patterns. The expression pattern of the 21 target genes in non‐immune organs, such as the lung or colon, and in non‐immune cells like adipocytes, questions the role of these organs and cell types in immune homeostasis and suggests additional, non‐immune functions of these molecules. The lack of impact of the *HLA* transcription factors studied here on *HLA* regulation in non‐immune tissues also supports a role for additional *HLA* transcription factors in these tissues. Finally, classical/non‐classical HLA classification based on molecule structure and genetic polymorphism does not seem to extend to their expression.

**Abbreviations**
IFNg: interferon gamma
MHC: major histocompatibility complex
MICA/B: MHC class I polypeptide‐related sequence A/B
RNAseq: RNA sequencing
RPKM: reads per kilobase per million mapped reads
sc: single‐cell
TNF: tumour necrosis factor

## 1 Introduction

HLA class Ib, MICA and MICB molecules deeply contribute to modulate inflammatory and allogenic responses. They are of considerable interest to narrow down diagnosis, adjust treatment and envision therapy for disorders involving immune surveillance.

HLA class Ib and MICA/MICB are major ligands of activating and inhibitory receptors mainly expressed by Natural Killer cells (NK), but also by CD8+ T, γδ T cells and B cells. NK are innate effector lymphocytes capable of cytotoxicity and cytokine secretion; they also participate in adaptative responses by regulating antigen‐presenting cells (APC) and the adaptive responses of T cells [1].

HLA‐G and HLA‐E tolerogenic function is driven by their association with LILRB1/2 and NKG2C/CD94 inhibitory receptors, respectively [2, 3, 4]. HLA‐F is also a ligand of inhibitory receptors (LILRB1, LILRB2, KIR3DL2, KIR3DL1), but displays its highest affinity for the KIR3DS1‐activating NK receptor [5, 6, 7]. MICA and MICB belong to the NKG2D ligands (NKG2DL), NKG2D being one of the most relevant NK cell‐activating receptors [8, 9].

Expressed as open conformers (OC) (i.e., dissociated from beta‐2 microglobulin and peptide), HLA class Ib and MICA/MICB may also have non‐immune functions like cell signalling, growth, differentiation, and cell communication, similarly to HLA class Ia molecules [10].

Abnormal expression of HLA class Ib and MICA/MICB is associated with the evolution of pathological conditions or clinical settings, such as viral infection, neoplasia development, or pregnancy outcome, but also in transplantation and graft success/rejection, whether expressed as the HLA‐peptide combination by APC or as an antigen per se by target cells [2, 3, 4, 11, 12, 13, 14, 15, 16, 17, 18, 19, 20, 21, 22, 23, 24]. Also, many studies explored the influence of *HLA class I* genetic polymorphisms on expression and disease progression: *HLA‐E* expression is strongly associated with its two main coding alleles [25, 26]; *HLA‐G* expression is linked to polymorphisms in its regulatory regions [27, 28] and *HLA‐F* expression is associated with non‐coding polymorphisms [29, 30, 31, 32, 33]. *MICA* and *MICB* both display polymorphisms [34]: while they do not correlate with *MICA* expression level [13, 35], variation in the *MICB* promoter was associated with its expression [36].

Despite considerable studies devoted to *HLA class Ib* and *MICA/MICB* genetics and their expression in pathological contexts, little information is available concerning their physiological expression and the magnitude of variation in different tissues and cell types, such data being limited to disparate case–control studies.

The constitutive transcriptional expression of *HLA class I* genes is driven by the NLRC5 transcription factor and to a lesser extent by CIITA, whereas *HLA* class *II* expression is reported to be activated by CIITA. INFg also plays a central role in *HLA* expression, directly by binding to promoter elements of *HLA‐E* and *HLA class Ia*, and indirectly as it regulates both *NLRC5* and *CIITA* expression [14, 37, 38, 39, 40, 41]. *MICA/MICB* transcription is induced by Sp1, Sp3 and Sp4 and NF‐Y transcription factors [36]. INFg and interleukin‐4 downregulate *MICA* expression through an upstream promoter (Lin, Hiron et al. 2018) [42], whereas TNFa upregulates *MICA* expression through NF‐Kb [43].

In physiological conditions, HLA‐E membrane‐bound expression is driven by an HLA class I peptide signal and is ubiquitously expressed at low levels on the cell surface of most tissues, with a higher expression reported in leukocytes, endothelial cells, and placental trophoblasts [2, 26, 44]. Physiological mechanisms leading to HLA‐F membrane expression remain unknown; HLA‐F protein was found at the cell membrane of migrating and invasive extravillous trophoblasts (EVTs) [45], surface epithelium, glandular epithelium, endometrium, and vascular formations [3]. Tissue‐restricted expression appears to be a hallmark of HLA‐G, as it is highly expressed by trophoblasts in the placenta and is also expressed in the thymus, cornea, pancreas, testis and endometrium, bronchial epithelial cells, mesenchymal stem cells, a few types of immune cells and erythroid and endothelial precursors [46, 47]. MICA and MICB are expressed intracellularly in most epithelia such as the gastrointestinal tract, breast, colon, liver, pancreas, stomach, bronchus, bladder, ureter and thymus epithelium [13].

Given their genetic similarity and common function, especially with regards to NK cell regulation, *HLA class Ia* and *HLA class Ib* may share some expression patterns in physiological conditions.

*HLA class Ia* transcriptional expression is regulated at the tissue level through an enhancer and silencer in the distal promoter, the CCAAT box for non‐lymphoid tissues, and the TATAA box for lymphoid tissues [12]. HLA class Ia expression occurs on the surface of nearly all nucleated cells, and that of HLA class II is restricted to both professional and non‐professional antigen‐presenting cells (APC) [14, 48, 49]. Few studies have analysed HLA class Ia and HLA class II differential expression according to tissues [12, 50, 51, 52]. Strong expression of HLA class Ia is observed in lymph nodes, spleen, gastrointestinal tract, respiratory and cardiovascular systems, male urogenital system (except in mature spermatozoa); absence or weak expression in the endocrine system (with the exception of adrenal), no expression in the brain (except in neurons and glia from different regions), and no expression in the pancreas, cornea or in trophoblasts [51, 52]. At the cell‐type level, HLA class Ia is observed in endothelial cells and fibroblasts. HLA class II expression is reported in lymphoid tissues, the gastrointestinal tract, but not in the colon, rectum or liver; in the respiratory and cardiovascular systems, in capillary endothelium, except in the brain and in the placenta. Finally, no expression is reported in the brain except in unidentified cells; no expression in the cornea or in trophoblasts [50].

Detailed *HLA class Ib* and *MICA/MICB* expression throughout the human body could help contextualise their study in clinical settings. Also, these expression data, when compared to those of *HLA class Ia, HLA class II* and their costimulatory molecules, would help gain insights into *HLA class Ib* and *MICA/MICB* biological functions and their role in homeostasis. Finally, the inclusion of the main HLA transcription factors in the dataset would help propose leads regarding their regulatory mechanisms.

With the advent of next‐generation sequencing and the availability of public sequencing data, experiments measuring overall RNA expression coupled with read mapping strategies across various cell types and tissues at resting state are now freely accessible to the scientific community [53]. Also, the remarkable advances in single‐cell analysis give the opportunity to study expression data at sub‐type cell level [54].

Correlation of protein and mRNA expression is subject to debate [55, 56, 57], still, RNAseq constitutes a pertinent measure in HLA and MICA/MICB analysis as it circumvents antibody technical limitations, differences in protein structure (combined with beta‐2 microglobulin and peptide, open conformer, combined with other molecules) or cellular compartment location (intracellular, membrane bound, shedding as soluble form or excreted via vesicles).

In this study we describe the physiological transcriptional expression of *HLA class Ib* and *MICA/MICB* throughout the human body and the different cell types. This analysis is matched with that of major *HLA* transcription factors and HLA costimulatory molecules involved in the immune response (CD40, CD80, CD86 and PD‐L1).

## 2 Material and Methods

### 2.1 Material

#### 2.1.1 Target Genes

Twenty‐one genes were selected for transcriptional expression analysis: six *HLA class I* and *class II* genes (*HLA‐A*, *‐B*, *‐C*, *‐DRB1*, *‐DQB1* and *‐DPB1*), seven *class Ib* genes (*HLA‐E*, *‐F*, *‐G*, *‐H* and *‐F‐AS1*, *MICA* and *MICB*), four genes encoding costimulatory HLA class II molecules (*CD80*, *CD86*, *CD40* and *PDL1*), and four genes encoding HLA transcription factors (*NLRC5*, *CIITA*, *TNF* and *INFg*).

#### 2.1.2 Tissue Expression Analysis

Transcriptional expression (RNA‐Seq) data for the target genes were obtained in 27 human organs and tissues from 95 samples from the ‘HPA RNA‐seq normal tissues’ project (BioProject PRJEB4337) from the National Centre for Biotechnology Information (NCBI) website [53, 54]. HPA project mRNA sequencing was performed on Illumina HiSeq2000 and 2500 machines from frozen tissue sections whose morphology was validated by a pathologist. Two to seven samples of each tissue or organ were analysed (Table S1). Quantification scores, akin to transcriptional expression, are expressed as ‘Count’ (total reads mapped to transcript features) and normalised as ‘RPKM’ (reads per kilobase per million mapped reads) to avoid biases due to transcript length. Quantification scores for the target genes are in Table S3.

#### 2.1.3 Single‐Cell Expression Analysis

Single‐cell expression for 18 of the 21 target genes (no data for *HLA‐H*, *‐AS*, and *PD‐L1*) was retrieved from the ‘Human Protein Atlas’ project (‘Downloadable data’ [58]). Single‐cell RNA sequencing (scRNA‐seq) data from 31 healthy human tissues were retrieved from publicly‐available genome‐wide studies, selected based on inclusion criteria ensuring data accuracy and reliability: sequencing for at least 4000 cells or no cell‐type pre‐enrichment. Cell type clusters corresponding to 15 different cell‐type groups in each tissue or organ are given in Table S2 [54]. Transcriptional expression normalised to transcripts per million protein coding genes and expressed as ‘nTPM’ are given in Table S6.

The HPA RNA‐seq and scRNA‐seq datasets share common organs and tissues (adipose tissue (body fat), bone marrow, brain, colon, endometrium, oesophagus, heart, kidney, liver, lung, lymph node, ovary, pancreas, placenta, salivary gland, skin, small intestine, spleen, stomach and testis) but some are unique to the HPA project (adrenal, appendix, duodenum, gall bladder, thyroid and urinary bladder) or to the scRNA‐seq analyses (breast, bronchus, eye, fallopian tube, PBMC, rectum, skeletal muscle, thymus, tongue and vascular tissue) (Tables S1 and S2).

### 2.2 Methods

Quantification score data in 27 human organs and tissues for 95 human samples from the ‘HPA RNA‐seq normal tissues’ project are expressed as RPKM and as Counts (Table S3), with median and range [min, max].

All association and correlation tests were performed with GRAPH PAD Prism 10.2.3 software (California USA). Pearson and Spearman tests were used for correlation analysis, with correction for multiple comparisons. Differences between two modalities were tested using a Mann–Whitney U test. A Kruskal–Wallis one‐way ANOVA followed by a Dunn post hoc test was used to test more than two modalities. Results were considered statistically significant for *p*‐values below 0.05. Principal component analysis (PCA) was used to summarise and visualise variation in gene expression for organs, tissues, or cell type groups.

scRNA‐seq data were used for descriptive analyses to define cell types that express HLA molecules. Since only merged data for each of the 31 tissue or organ samples are available, no statistical analyses were performed on scRNA‐seq data. Transcriptional expression of each gene according to cell type clusters and cell types are given in Table S6.

## 3 Results

### 3.1 *HLA‐E* and *‐H* Display High Expression Levels Characteristic of *HLA Class Ia* Genes

**FIGURE 1:**
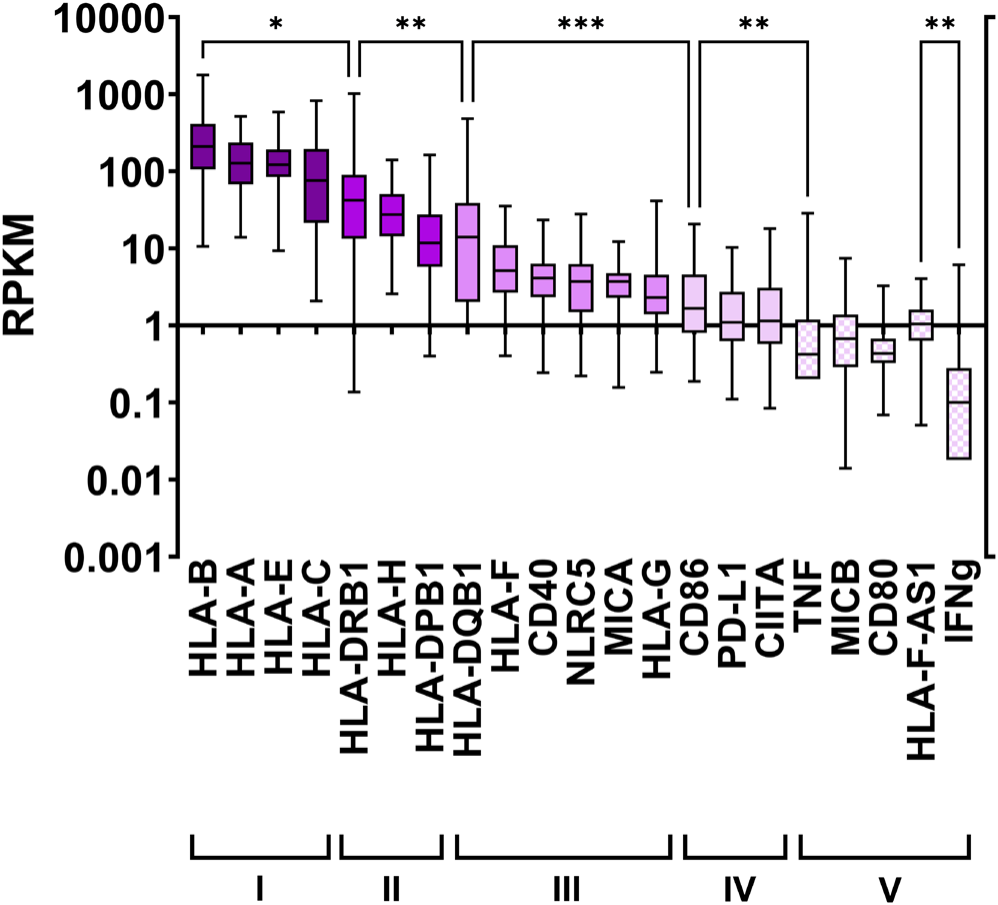
Expression levels for the 21 *HLA* and *HLA‐related* genes investigated fall into five groups. mRNA expression for the 21 target genes in 95 individuals is quantified as reads per kilobase per million reads placed (RPKM) and presented as box and whiskers (min to max) with a line at the median. A Log_10_ scale is used. Genes are sorted from left to right based on their magnitude of expression (median). Five statistical groups were defined through pairwise comparisons: for each group there is no statistical difference in expression between the gene with the highest expression and the gene with the lowest expression (*p*‐values are given in Table S4). Each group has a unique colour and the group number is given under the gene names. The level of confidence for the statistical differences between the highest values of each group is given with asterisks (Kruskal–Wallis test; one asterisk, a = 0.05, two asterisks, a = 0.01 and three asterisks a = 0.001). *INFg* was included in group V, even though its expression is significantly lower than the other genes of the group.

Expression for 21 *HLA* and *HLA‐related* genes was assessed using data from the HPA RNA‐seq normal tissues project (Figure 1 and Table S4): these include six *HLA class Ia* and class *II* genes (*HLA‐A, ‐B, ‐C*, *HLA‐DRB1, HLA‐DQB1* and *HLA‐DPB1*), seven *HLA class Ib* genes (*HLA‐E, ‐F, ‐G, ‐H, ‐F‐AS1* and *MICA‐B*), four genes encoding costimulatory HLA class II molecules (*CD80, CD86, CD40* and *PDL1*), and four genes encoding HLA transcription factors (*NLRC5, CIITA, TNF* and *INFg*).

When all tissues are considered together, median gene expression differs by orders of magnitude and hence falls into five statistically distinct groups: *HLA‐A, ‐B, ‐C*, and *HLA‐E* belong to the group with the highest expression (group I), *HLA‐H, ‐DRB1* and *‐DPB1* form the second highest expression group (group II), while *HLA‐F, ‐DQB1, ‐G, MICA, CD40* and *NLRC5* form the third group (group III). The remaining eight genes (*CD80, CD86, PD‐L1, CIITA, TNF, MICB, INFg* and *HLA‐F‐AS1*) are less expressed and form groups IV and V (Figure 1).

This first analysis hence shows that while five of the six classical *HLA* genes belong to the two groups with highest expression (groups I‐II), two non‐classical *HLA* genes also belong to these two groups (*HLA‐E* and *‐H*) and the classical/non‐classical distinction is thus not absolute with regards to overall expression.

### 3.2 Gene Expression Correlation and Distribution Challenges HLA Classification

**FIGURE 2:**
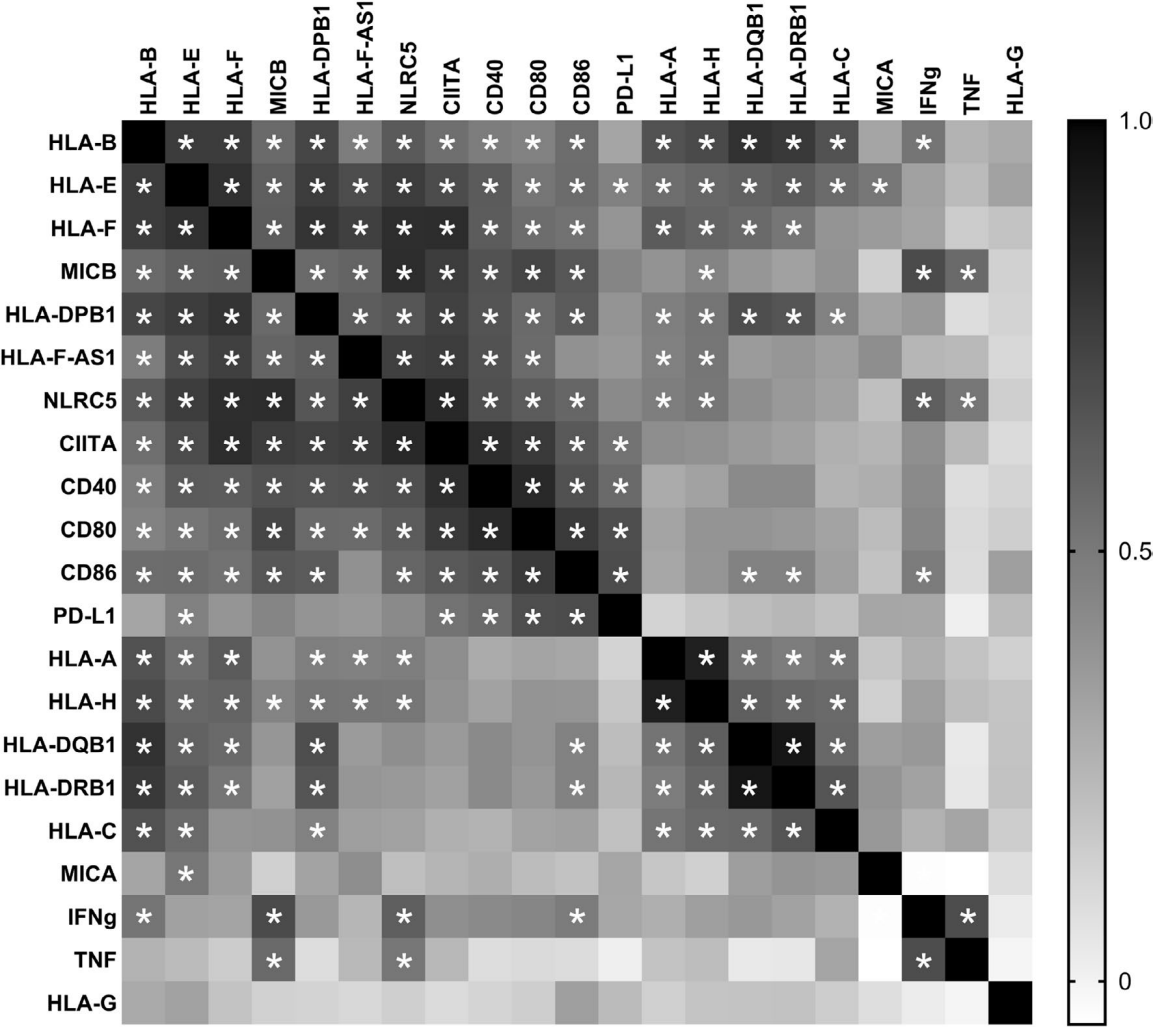
Expression patterns for the 21 *HLA* and *HLA‐related* genes investigated are significantly correlated for two blocks of genes representing a total of 16 genes. Correlation matrix heatmap between mRNA expression of 21 genes in 95 individuals. Genes are organised according to their correlation values. Asterisks correspond to adjusted *p*‐values < 0.01 (given in Table S5C). The first block includes *HLA‐B* to *CD86* (11 genes) and the second *HLA‐A*, *‐B*, *‐C*, ‐*E*, *‐F*, ‐H, DRB1, ‐DQB1 and *‐DPB1* (nine genes).

The patterns of expression of the 21 studied genes were compared using a correlation matrix of gene expression for all the investigated tissues (Figure 2 and Table S5).

Two sets emerge from this analysis; the first block is the largest with 11 genes for which all but one of the pairwise comparisons (*CD86* vs. *HLA‐F‐AS1*) show statistically significant correlations. This first set includes a mix of *HLA class Ia* genes (*HLA‐B* and *‐DPB1*), *HLA class Ib* genes (*HLA‐E, ‐F* and *‐F‐AS1, MICB)*, genes encoding costimulatory HLA class II molecules (*CD80, CD86* and *CD40*), and genes encoding HLA transcription factors (*NLRC5* and *CIITA*). These 11 genes encompass all expression groups (Figure 1) and hence show a qualitative correlation despite the various levels of expression.

The second set includes all six *HLA class Ia and class II* genes and three *HLA class Ib* genes (*HLA‐E, ‐F, ‐H*), and all pairwise comparisons but one (*HLA‐C* vs. *HLA‐F*) are statistically significant. These nine genes display the highest expression (Figure 1) and four of them also belong to the first block (*HLA‐B*, *‐E*, *‐F* and *‐DPB1*). That the same four genes are represented in the two blocks, but the remaining seven genes of set #1 and five genes of set #2 show limited correlations (seven significant correlations out of 35) shows that there is some asymmetry in the correlations. Analysis of individual expression patterns shows that this asymmetry stems from divergence in tissue‐specific expression (as described in detail in the subsequent sections).

Finally, some genes distinguish themselves by limited correlations to one set (*PDL‐1* with other genes encoding costimulatory HLA class II molecules), to a handful of genes (*INFg* with *HLA‐B, MICB, NLRC5* and *CD86*; *TNF* with *MICB* and *NLRC5*; *MICA* with *HLA‐E*) or with no statistically significant correlation (*HLA‐G*).

The analysis hence shows that 16 of the 21 genes studied show some correlated expression in two major sets. In particular, the largest of the two sets shows that the correlation extends to members of all the functional and expression‐level groups. That both sets include both classical and non‐classical *HLA* genes also shows that expression patterns are not specific to either group. Furthermore, this analysis highlights quite remarkable specific, or even unique, expression patterns for some of the *HLA* and *HLA‐related* genes.

### 3.3 *HLA* and *HLA*‐Related Genes Fall Into Groups With Tissue‐Specific Expression

While the overall analyses show correlated expressions for a large group of *HLA* and *HLA‐related* genes (Figure 2), analysis of individual expression patterns in tissues and organs shows tissue‐specificity (Figures S1 and S2), as suggested by the large variances in Figure 1. For example, *HLA class Ia* and *class Ib* genes (except *HLA‐G*) display their highest expression in the lung, spleen, lymph node, bone marrow, colon, small intestine and appendix, while there is virtually no *HLA class I* expression in the pancreas. *HLA‐G* distinguishes itself by its high expression in the placenta, colon, lung and secondary lymphoid tissues.

*MICA* is also broadly expressed, displaying highest expression in the lung, endometrium, spleen, thyroid and gall bladder, and lowest expression in bone marrow, liver, salivary gland, brain and pancreas. Conversely, *MICB* is mainly expressed in lymph nodes, bone marrow, spleen and appendix (Figures S1 and S2).

*HLA class II* genes display similar expression patterns: *HLA‐DRB1* and *‐DQB1* have their highest expression in the lung, lymph node, spleen, urinary bladder and stomach. *HLA‐DRB1* is also highly expressed in the appendix, small intestine, gall bladder and colon. *HLA‐DPB1* displays highest expression in the spleen, lymph node, lung and appendix.

HLA class II costimulatory molecules (*CD40, CD80, CD86* and *PD‐L1*) show differences in tissue expression: *CD40* is the most widely expressed, with the highest expression in secondary lymphoid organs and in the lung; intermediate expression in all other organs and tissues, whereas the testis, pancreas, brain, and bone marrow display weak expression. *CD86* has an expression level equivalent to that of *CD40* but is more restricted: high expression is observed in the appendix, lymph node, spleen, gall bladder, placenta, lung and urinary bladder. *CD80* displays low expression, mainly in the appendix, lymph node, spleen and lung. *PD‐L1* has its highest expression in the appendix, placenta, spleen, lung, heart, lymph nodes and gall bladder.

*NLRC5* is highly expressed in the spleen, lymph nodes, bone marrow and appendix. It displays an intermediate expression in other organs and is weakly or not expressed in the pancreas, liver, kidney, brain, testis and ovary and placenta. *CIITA* is highly expressed in the spleen, lymph node and appendix, and shows intermediate expression in the lung, small intestine, duodenum, gall bladder, urinary bladder and stomach.

Finally, expression of *TNF* and *INFg* is restricted: *TNF* is highly expressed in bone marrow and displays intermediate levels in lymph nodes, the appendix and lung. *INFg* has a high expression in bone marrow, lymph nodes and the appendix.

### 3.4 *HLA* and *HLA*‐Related Gene Expression in the Lung is Equivalent to That of Lymphoid Organs

**FIGURE 3:**
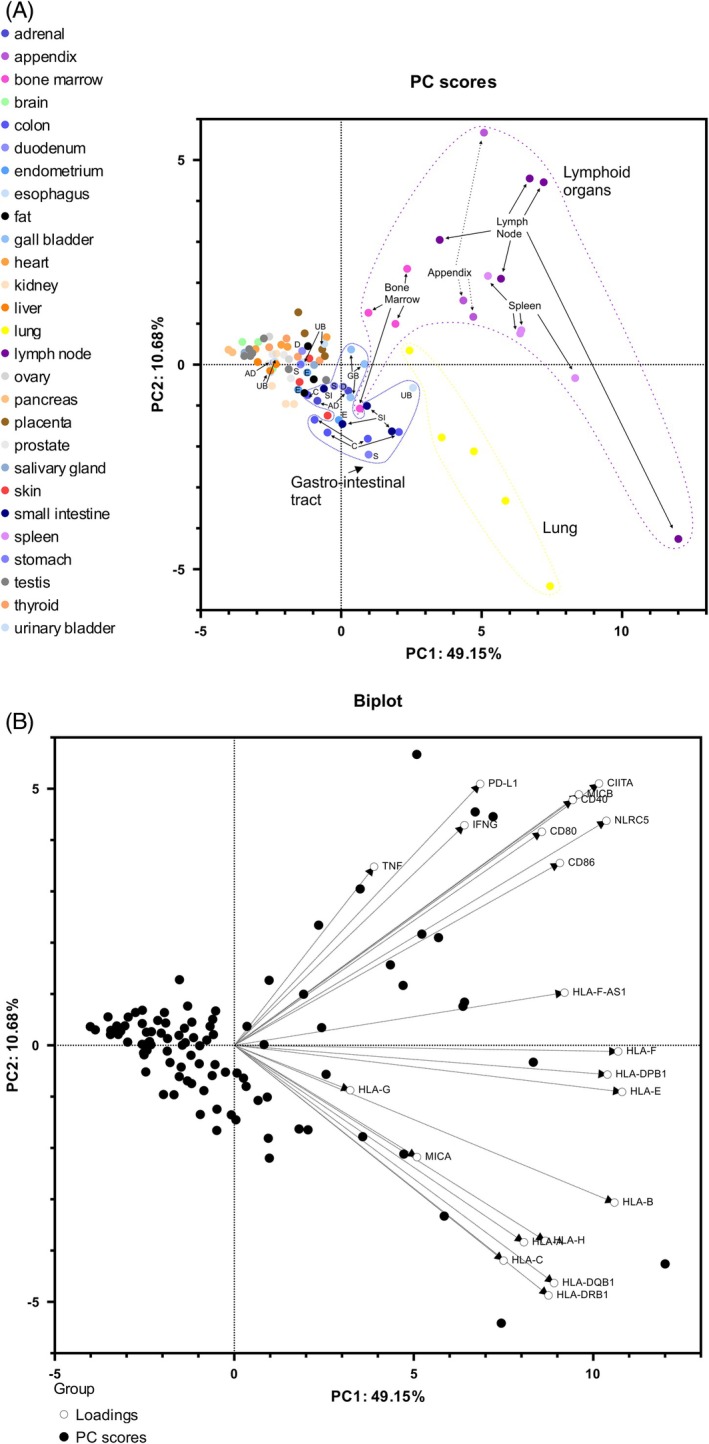
Principal component analysis (PCA) for the expression of the 21 target genes in 95 tissues separates lymphoid organs, lungs, and gastro‐intestinal tract organs from the other tissues. Principal components 1 and 2 represent, respectively, 49.15% and 10.68% of variance. Tissues are plotted by colour; for the gastro‐intestinal tract, letters identify the different tissues (A**)**. PCA biplot illustrates the loadings of each variable (B).

The expression patterns of the 21 studied genes in the 27 tissues and organs of the HPA project were further investigated by Principal Component Analysis (PCA). The first two components, PC1 and PC2, account for 49.15% and 10.68% of the total variance, respectively (Figure 3A).

Three organs stand out in this PCA with distinct spatial distributions: the lymphoid organs, the lung, and the gastro‐intestinal tract organs. Analysis of the PCA biplot, that includes the loadings of each variable, shows that PC1 separates gut, lung and lymphoid organs from other organs according to the expression of all studied genes but with the lowest impact coming from the five genes showing limited correlations with the other 16: *TNF*, *HLA‐G*, *MICA*, *INFg* and *PD‐L1* (Figure 3B). Consistent with this, when the PCA is performed after excluding these five genes, the proportion of variance explained by PC1 increases to 59.26% (data not shown). PC2 distinguishes lymphoid organs from the rest and is more specifically influenced by costimulatory HLA class II molecules, *HLA* transcription factors, and *MICB* on the upper side, and by *MICA*, *HLA‐A*, *‐B*, *‐C*, *‐H*, *‐DRB1*, *‐DQB1* and to a lesser extent by *HLA‐G* on the lower side.

Individual gene expressions show that the three sets of organs are those where the target genes are the most expressed (Figure S1), with PC1 correlating with the differences in expression. Thus, strikingly, the lung displays an expression of *HLA* and *HLA‐related* genes as high as that of primary and secondary lymphoid tissues. Gastro‐intestinal tract organs also highly express the 21 studied genes, although to a lesser extent than lymphoid organs and the lung (Figure 3A).

### 3.5 Single‐Cell Expression: Non‐Immune Cells Highly Express *HLA Class Ib* and *MICA*

Expression patterns for 18 of 21 studied genes (no data for *HLA‐F‐AS1*, *HLA‐H* and *PD‐L1*) were analysed at cellular level using single cell RNA‐seq data (Table S6; expression in the different cell type groups, as defined in Table S2 is in Figure S3).

Strikingly, this analysis shows that adipocytes, endothelial cells, and immune cells have unexpectedly close expression patterns, with high expressions of *HLA class Ia* and *class II*, *HLA‐F, HLA‐E* and *CD40*. Immune cells distinguish themselves, however, by high expressions of *MICB, CD86, TNF* and *INFg*.

Among the different cell type groups and when considering all tissues, *HLA* and *HLA‐related* genes display different expression patterns. A first group is made of *HLA‐A, ‐B, ‐C*, *‐E* and *‐F* that are highly expressed in immune cells, adipocytes and endothelial cells but are not or are weakly expressed in neuronal cells, germ cells, and trophoblasts, and at an intermediate level in other cell type groups. *HLA‐G* has a unique profile, being highly expressed by trophoblasts and at a very low level, if any, in adipocytes, glial cells, neuronal cells, squamous epithelial cells, endothelial cells or immune cells.

*MICA* and *MICB* also show different patterns of expression: *MICA* is more expressed in adipocytes, pigment, endothelial, and mesenchymal cells, not expressed in trophoblasts, and weakly expressed in neuronal and germ cells; other cell type groups display intermediate levels. Notably, MICA is absent from platelets and neutrophils. Conversely, *MICB* is mainly expressed by immune cells.

*HLA class II* genes are highly expressed in immune cells, glial cells, and adipocytes, at low levels in endothelial cells, glandular and specialised epithelial cells, and pigment cells, and are absent from other cell type groups, especially neuronal, trophoblast, and germ cells.

CD40 is broadly expressed, with the highest levels in adipocytes, endothelial and immune cells, whereas it is not observed in neuronal cells and trophoblasts. CD80 is observed in immune cells, and CD86 is observed in immune cells and glial cells.

*NLRC5* is mainly expressed in immune cells, adipocytes and endothelial cells; other cell types showed intermediate expression, whereas no expression is reported in trophoblasts, germ cells or neuronal cells. *CIITA* is mainly expressed in immune cells, glial cells, adipocytes, endothelial cells and pigment cells, and not or weakly expressed in other cell types.

*TNF* and *INFg* are mainly expressed in immune cells; both are weakly expressed in adipocytes, endothelial cells, and pigment cells. TNF expression is also observed in squamous and glandular epithelial cells and glial cells.

## 4 Discussion

HLA class Ib and MICA/MICB are major ligands of regulatory receptors expressed by immune effector cells. Their expression is broadly associated with the outcome of many diseases; however, little is known regarding their physiological expression in the different tissues, organs and cell types.

Our main findings are that (i) *HLA‐E* and *HLA‐H* are highly expressed, with levels equivalent to those of *HLA class I*a and *HLA class II*, (ii) *MICA* and *MICB* display distinct expression patterns, *MICB* being specific to lymphoid tissues, (iii) *HLA‐B* expression pattern challenges its classification as an *HLA class Ia* gene, (iv) *HLA class II* genes are widely expressed, (v) the lung and adipocytes, classified as non‐immune actors, highly express *HLA* genes.

Transcriptional expression was retrieved from two projects based on RNA sequencing (RNA‐seq) and single‐cell (sc) analysis. Single‐cell sorting technologies, coupled with RNA‐seq analysis, offers enhanced access to in‐depth transcriptomics information using cell type identification [59]. RNA‐seq provides a highly sensitive and resolutive solution to explore gene expression and enables comparison via quantitative analysis. A tissue or organ bulk approach provides global information regardless of cell heterogeneity. However, sequencing quality is dependent on sample panel quality, and artefacts and bias may be observed [60, 61]. In particular, with many of the genes studied here being stress inducible, expression could be impacted by sample collection and processing. Limiting this risk here, samples were collected under controlled conditions that would limit cellular stress [53].

Genetic variation was not considered in this study, although expression levels can be impacted by genetics [26, 60, 61], in particular for HLA molecules [12, 62, 63, 64, 65]. In the original description of the source data used here, Fagerberg et al. [53] pointed out the high correlation between samples from the same tissues in different individuals, supporting low biological inter‐individual variability. No inter‐individual comparison was sought in this study, and so any potential variation in allelic expression is taken into account and integrated in the standard deviations. Similarly, post‐transcriptional mechanisms, such as alternative splicing or RNA silencing [14, 66], are not distinguished in the primary data.

Our analysis showed that nearly all tissues, immune and non‐immune cells express *HLA‐E*, *HLA‐H* and *HLA‐F*, and that *HLA‐E* and *HLA*‐*H* are the most expressed, with expression levels similar to those of *HLA class Ia*.

Previous observations of *HLA‐E* and *HLA‐F* physiological expression in the different tissues were globally confirmed [2, 26, 44].

*HLA‐H*, categorised as one of the many *HLA class I* pseudogenes notably because of the shortened reading frame of most of its alleles, was reported as having a role in HLA‐E mobilisation at the cell surface [67]. Whether *HLA‐H* plays a role in immune homeostasis or whether its high expression is linked to mutual *HLA‐A* regulatory mechanisms is not clear.

*HLA‐G* distinguishes itself with a relatively restricted expression pattern and thus displays low correlation with other *HLA* genes. Consistent with previous studies, *HLA‐G* expression is confirmed in the placenta, colon, lung, testis, and endometrium, but is virtually absent in the eye and pancreas [46, 47]. At the cell type level, *HLA‐G* is expressed at low levels by immune cells, endothelial cells, and was not observed in mesenchymal stem cells [46, 47].

Concerning *MICA/MICB*, our analyses show that *MICA* is expressed at low levels by almost all organs and cell types, and distinguishes itself by its poor correlation with the expression patterns of other *HLA* genes, notably with the transcription factors included in the analysis. *MICB* expression is restricted to lymphoid organs and immune cells, and is highly correlated with *TNF* and *IFNg*. Thus, our data highlight the differences between these two NKG2D ligands, one being mostly expressed by non‐immune cells, the second being immune‐tissue‐specific. Many studies on MICA/MICB merged expression data from both ligands since validated antibodies are directed against the alpha 3 domain of both molecules [13, 68]. The finding that *MICA* and *MICB* are expressed by distinct cell types and seem regulated by different pathways highlights the central role of these ligands in NK cell regulation through the NKG2D receptor in all tissues.

We confirm *that HLA class Ia* is expressed in almost all tissues, as reported in previous studies [14, 51], notably with high expression in primary and secondary lymphoid organs, the respiratory system and gastrointestinal tract.

Based on expression patterns, our results support that *HLA‐B*, whose transcription level is the highest, is closer to *HLA‐E, HLA‐F, MICB* and *HLA‐DPB1* than to *HLA‐A* or *HLA‐C*. This peculiar profile of *HLA‐B*, close to that of inducible molecules at the cell surface, questions its function(s) and regulatory mechanism(s).

Based on correlation analyses, *NLRC5* and *CIITA* transcription factors are seen to be deeply involved in *HLA‐E, ‐F* and *MICB* expression. Both transcription factors are also more correlated to *HLA‐B* than to *HLA‐A or HLA‐C. C*onversely, they do not seem to be major transcription factors of *MICA* and *HLA‐G*. *IFNg* and *TNF*, in regard to their expression being mainly restricted to immune cells, are highly correlated to *MICB*.

*HLA class II* transcripts, whose expression pattern is less consensual [49, 50], are observed in a large number of organs, such as the respiratory system and gastrointestinal tract, and lymphoid organs and cells, including glial cells which are involved in many immune and non‐immune functions [69, 70, 71, 72, 73].

Our study highlights that, at the organ level, striking expression patterns were observed in the lung, with expression levels of all of the genes studied as high as those of primary and secondary lymphoid organs, including by non‐immune cells such as endothelial, mesenchymal or specialised epithelial cells. To a lesser extent, the colon and small intestine also display high expression of these genes. The tissues and organs of the gut‐lung axis are well described for their central role in controlling the complex immune responses preventing pathogen infection and tolerating the commensal microbiome [4, 14, 26]. RNA constitutive expression in many cell types and tissues may constitute a reservoir of ready‐to‐use molecules for the cells to rapidly express membrane‐bound and/or released HLA and adapt the immune cells response, especially those of NK cells whose activation depends on the balance between signals induced by ligands binding activating or inhibitory receptors. The high expression of *HLA class Ib* in the lung and colon further confirms the interest of these molecules as biomarkers or therapeutic targets in the pathologies affecting these organs, such as cancer, inflammatory diseases or in transplantation therapy.

Of note, *NLRC5* and *CIITA* display intermediate expression in lung, whereas *HLA* expression is high, supporting specific regulatory mechanisms in these organs.

At cell‐type level, our study reveals that adipocytes display as high expression of *HLA class Ia, class Ib* and *HLA class II* molecules (except *HLA‐G* and *MICB)* as immune cells. As in lung, *NLRC5* and *CIITA* display intermediate expression, further supporting alternative *HLA class I* regulatory mechanisms. Adipocytes encompass different types of cells, illustrated by their distinct precursor cells and their broad distribution in the human body. Accordingly, adipocytes embrace a wide diversity of functions that include both immune cell interactions and immune role per se. Adipocytes produce a wide range of pro‐ and anti‐inflammatory molecules (cytokines, hormones, lipids) and vesicles modulating inflammation and immune cell activity and are able to present antigens [74, 75]. The observation of high expression of all *HLA* and *MICA* genes under study (except *HLA‐G*) as well as *CD40*, but neither *CD80* nor *CD86* in these cells further supports their central role in immunological processes, with an expression profile in favour of immune activation mechanisms. Conversely, the non‐immune function of these molecules cannot be ruled out; HLA class Ib may display non‐immunological functions, as demonstrated by the role of HLA‐F in cell proliferation [76]. HLA class Ia molecules, expressed as open conformers (OC), are involved in other functions than immune response such as cell signalling, growth, differentiation and cell communication [10]. Induced by several physiological settings such as metabolic state and nutritional needs, HLA class I expressed as OC interact with non‐immune receptors for hormones, growth factors, cytokines and neurotransmitters [10]. In non‐immune cells, the low expression of *TNF* and *INFg* as compared to the high level of *HLA* expression supports distinct mechanisms ruling *HLA* regulation and potentially non‐immune functions.

In conclusion, *HLA*‐*E*, *‐F*, *‐H* and *MICA* genes are broadly expressed throughout the human body in physiological conditions, whereas *MICB* is restricted to immune organs and cells. More generally, the expression pattern of the 21 target genes in non‐immune organs such as the lung or colon, and in non‐immune cells like adipocytes, questions the role of these organs and cell types in immune homeostasis and suggests additional, non‐immune functions of these molecules. The lack of impact of the HLA transcription factors studied here (*NLRC5, CIITA, TNF* and *INFg*) on HLA regulation in non‐immune tissues also supports a role for additional HLA transcription factors in these tissues. Finally, classical/non‐classical HLA classification based on molecule structure and genetic polymorphisms does not seem to extend to their expression, especially for *HLA‐B*.

## Conflicts of Interest

The authors declare no conflicts of interest.

## Supporting information


**Figure S1:** Mean mRNA expression for 21 target genes in 27 different tissues (*N* = 95) analysed in the ‘HPA RNA‐seq normal tissues’ project (values are expressed as reads per kilobase per million reads placed, RPKM). S1A: *HLA‐H*, *‐G* and *MICA*. S1B: *HLA‐F*, *‐F‐AS1* and *MICB*. S1C *HLA‐E*, *‐A*, *‐B*, and *‐C*. S1D: *HLA‐DRB1*, ‐*DQB1*, and *‐DPB1*. S1E: *CD40*, *CD80*, *CD86* and *PD*‐*L1*. S1F: *NLCR5*, *CIITA*, *INFg* and *TNF*.

**Figure S2:** Highlights of tissue‐specific gene expression that illustrates asymmetric correlation revealed in Figure 2.

**Figure S3:** Single‐cell RNA sequencing (scRNA‐seq) data for 18 of the 21 target genes in 31 healthy human tissues. Transcriptional expression is normalised to transcripts per million protein coding genes and expressed as ‘nTPM’. S3A: *HLA‐F*, *‐G*, *MICA* and *MICB*. S3B: *HLA‐E*, *‐A*, *‐B* and *‐C*. S3C: *HLA‐DRB1*, *‐DQB1* and *‐DPB1*. S3D: *CD40*, *CD80* and *CD86*. S3E: *NLCR5*, *CIITA*, *INFg* and *TNF*.

**Table S1:** Number of samples for each human organ and tissue from the ‘HPA RNA‐seq normal tissues’ project used for RNA‐seq analyses. Presence or absence of these organs and tissues in the scRNA‐seq project is indicated.
**Table S2:** Cell type clusters corresponding to 15 different cell type groups used for single‐cell RNA sequencing (scRNA‐seq) in 31 healthy human tissues. Presence or absence of these tissues in the ‘HPA RNA‐seq normal tissues’ project is indicated.
**Table S3:** mRNA expression for the 21 target genes in 95 individuals of the ‘HPA RNA‐seq normal tissues’ project. Values are expressed as reads per kilobase per million reads placed (RPKM) and approximate total reads mapped to gene transcript features (Count). Median, minimum, maximum and standard deviation are given for each gene.
**Table S4:** Comparison of mRNA expression levels for the 21 target genes (Kruskal–Wallis one‐way ANOVA followed by a Dunn post hoc test). Plain and dash line squares highlight expression levels that are not significantly different.
**Table S5:** Correlation values. (A), *p*‐values of correlation tests (B) and adjusted *p*‐values (C) for the mRNA expression patterns (‘HPA RNA‐seq normal tissues’ project) of the 21 target genes (Pearson test; simple correction for multiple comparisons).
**Table S6:** scRNA‐seq results for 18 of the 21 target genes (no data for *HLA‐F‐AS1*, *‐H* and *PD‐L1*) in different cell‐type groups in each tissue or organ [53]. Transcriptional expression is normalised to transcripts per million protein coding genes and expressed as ‘nTPM’.

## Data Availability

The data that support the findings of this study are available in BioProject PRJEB4337 at https://www.ncbi.nlm.nih.gov/bioproject?term=PRJEB4337&cmd=DetailsSearch, reference number 52, 53. These data were derived from the following resources available in the public domain: HPA RNA‐seq normal tissues’, https://www.ncbi.nlm.nih.gov/bioproject?term=PRJEB4337&cmd=DetailsSearch.
