# Supplementary figures and images for: HLA Class Ib and MICA/MICB Expression in Human Tissues and Cell Types: Reshuffling Immune Players
Source: HLA. 2025 Sep 10;106(3):e70390. doi: 10.1111/tan.70390 (PMC12422173; doi:10.1111/tan.70390)

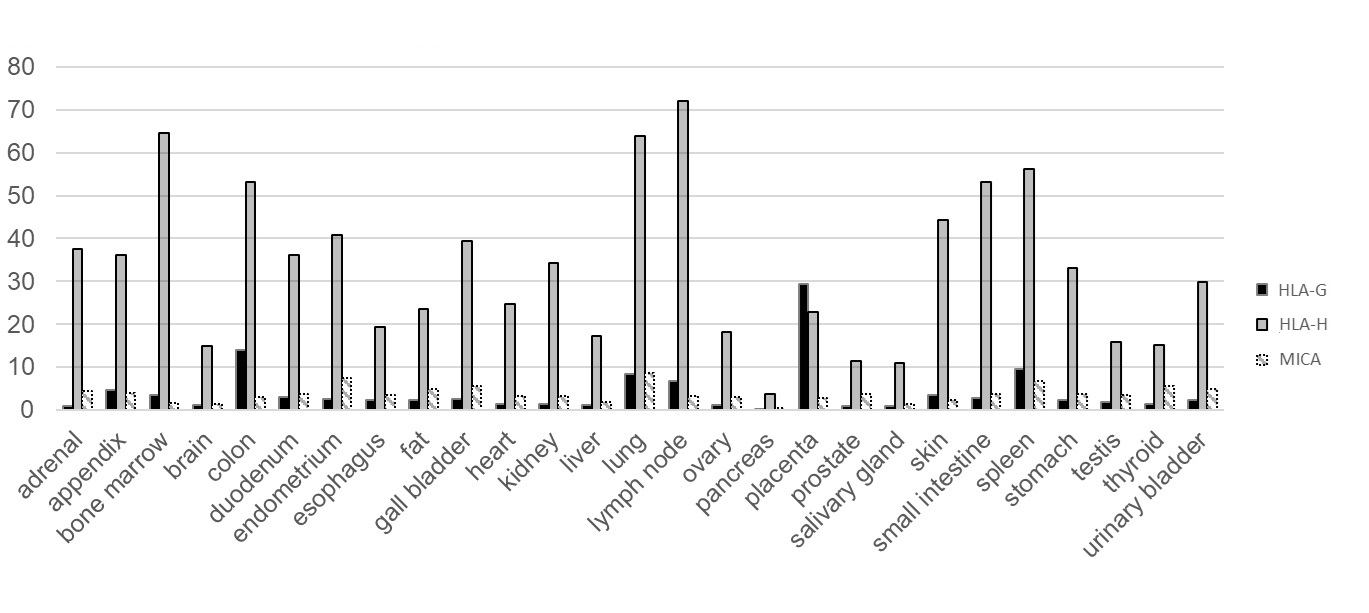

Supplement: Supplementary file 1 — Figure S1: Mean mRNA expression for 21 target genes in 27 different tissues (N = 95) analysed in the ‘HPA RNA‐seq normal tissues’ project (values are expressed as reads per kilobase per million reads placed, RPKM). S1A: HLA‐H, ‐G and MICA. S1B: HLA‐F, ‐F‐AS1 and MICB. S1C HLA‐E, ‐A, ‐B, and ‐C. S1D: HLA‐DRB1, ‐DQB1, and ‐DPB1. S1E: CD40, CD80, CD86 and PD‐L1. S1F: NLCR5, CIITA, INFg and TNF. [file TAN-106-e70390-s002.zip › tan70390-sup-0002-FigureS1@SupFig1A.jpg]

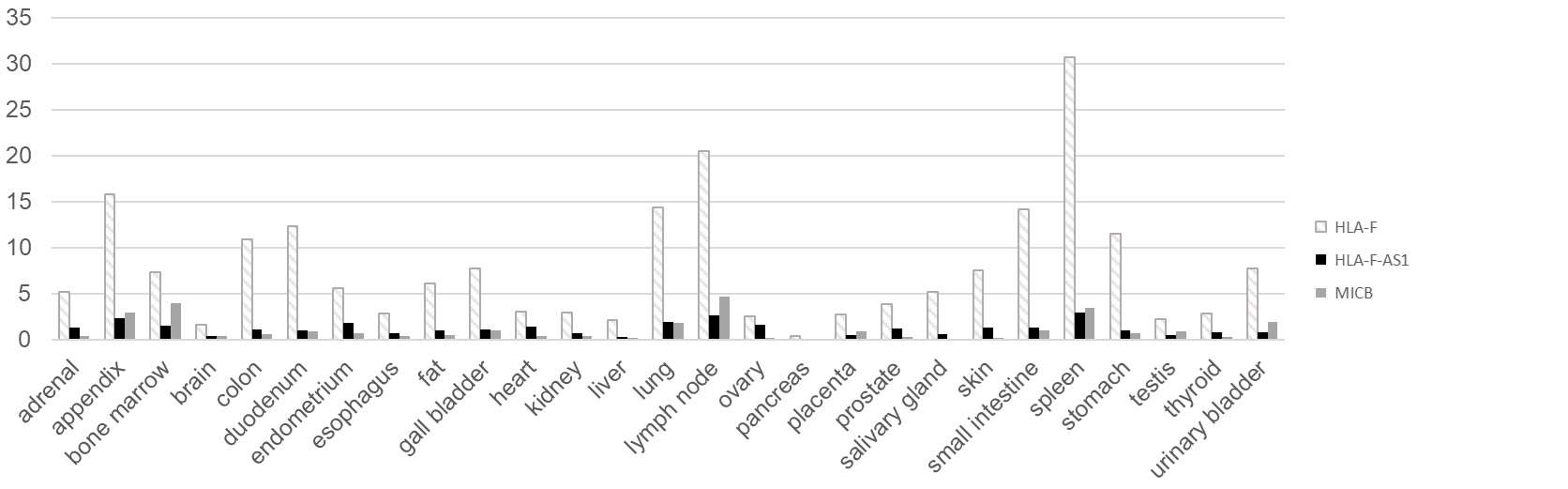

Supplement: Supplementary file 1 — Figure S1: Mean mRNA expression for 21 target genes in 27 different tissues (N = 95) analysed in the ‘HPA RNA‐seq normal tissues’ project (values are expressed as reads per kilobase per million reads placed, RPKM). S1A: HLA‐H, ‐G and MICA. S1B: HLA‐F, ‐F‐AS1 and MICB. S1C HLA‐E, ‐A, ‐B, and ‐C. S1D: HLA‐DRB1, ‐DQB1, and ‐DPB1. S1E: CD40, CD80, CD86 and PD‐L1. S1F: NLCR5, CIITA, INFg and TNF. [file TAN-106-e70390-s002.zip › tan70390-sup-0003-FigureS1@SupFig1B.jpg]

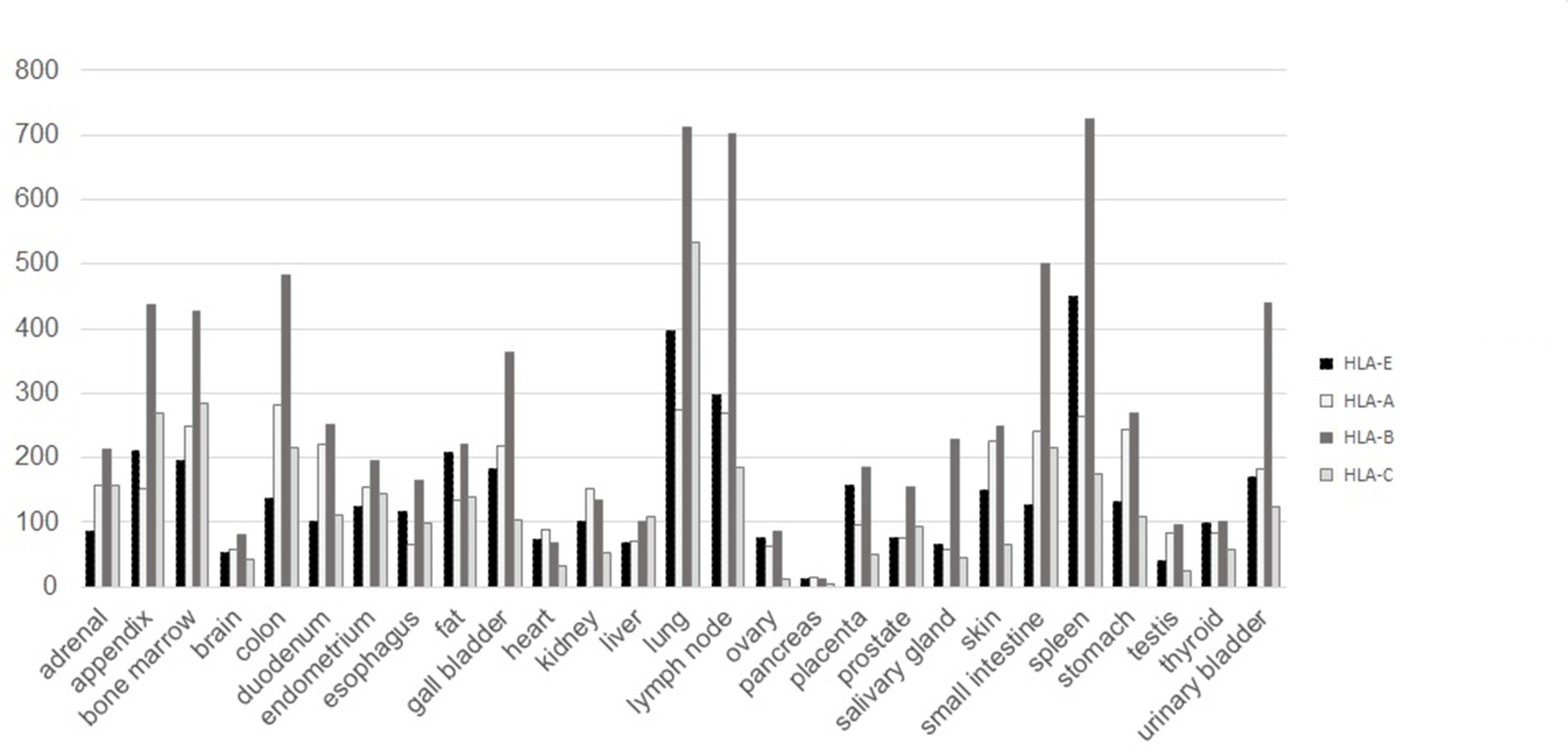

Supplement: Supplementary file 1 — Figure S1: Mean mRNA expression for 21 target genes in 27 different tissues (N = 95) analysed in the ‘HPA RNA‐seq normal tissues’ project (values are expressed as reads per kilobase per million reads placed, RPKM). S1A: HLA‐H, ‐G and MICA. S1B: HLA‐F, ‐F‐AS1 and MICB. S1C HLA‐E, ‐A, ‐B, and ‐C. S1D: HLA‐DRB1, ‐DQB1, and ‐DPB1. S1E: CD40, CD80, CD86 and PD‐L1. S1F: NLCR5, CIITA, INFg and TNF. [file TAN-106-e70390-s002.zip › tan70390-sup-0004-FigureS1@SupFig1C.jpg]

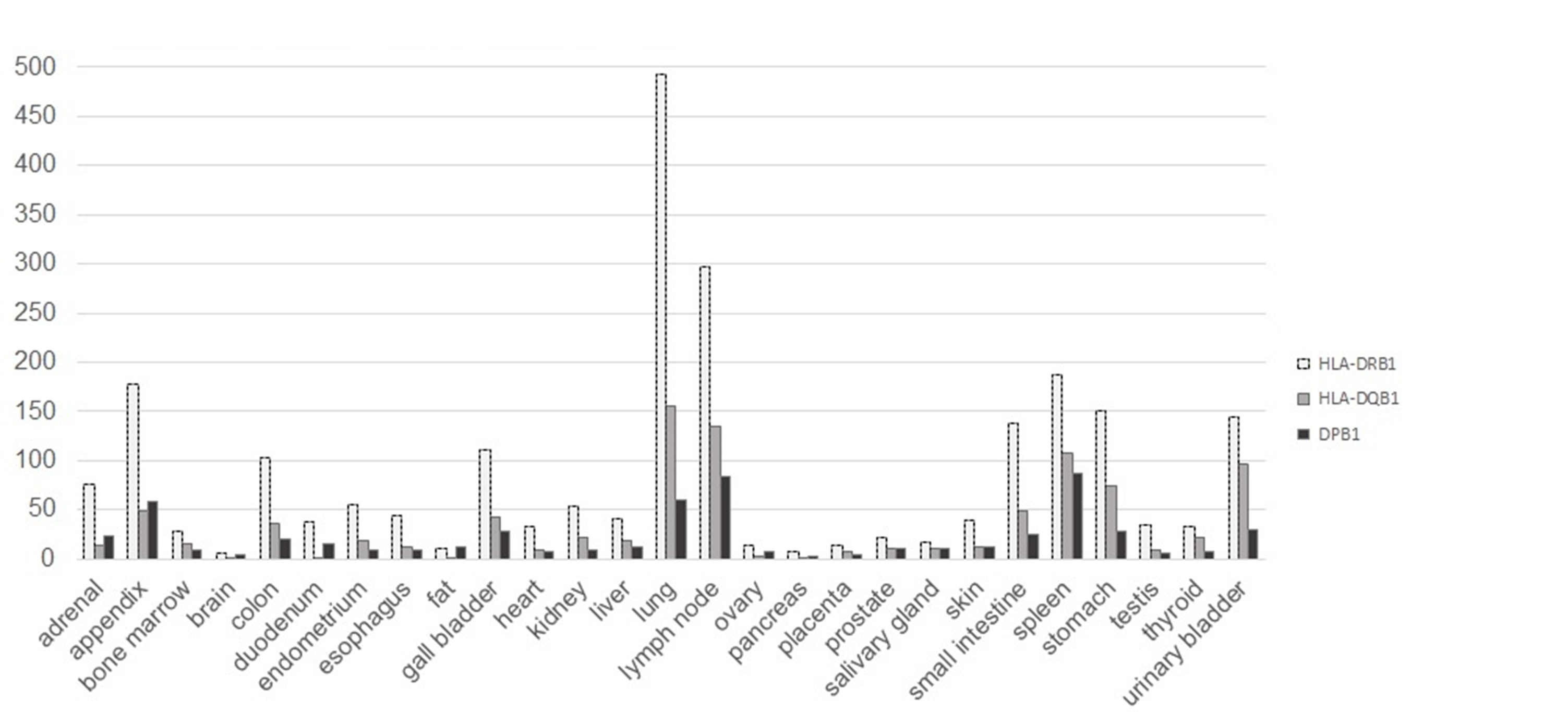

Supplement: Supplementary file 1 — Figure S1: Mean mRNA expression for 21 target genes in 27 different tissues (N = 95) analysed in the ‘HPA RNA‐seq normal tissues’ project (values are expressed as reads per kilobase per million reads placed, RPKM). S1A: HLA‐H, ‐G and MICA. S1B: HLA‐F, ‐F‐AS1 and MICB. S1C HLA‐E, ‐A, ‐B, and ‐C. S1D: HLA‐DRB1, ‐DQB1, and ‐DPB1. S1E: CD40, CD80, CD86 and PD‐L1. S1F: NLCR5, CIITA, INFg and TNF. [file TAN-106-e70390-s002.zip › tan70390-sup-0005-FigureS1@SupFig1D.jpg]

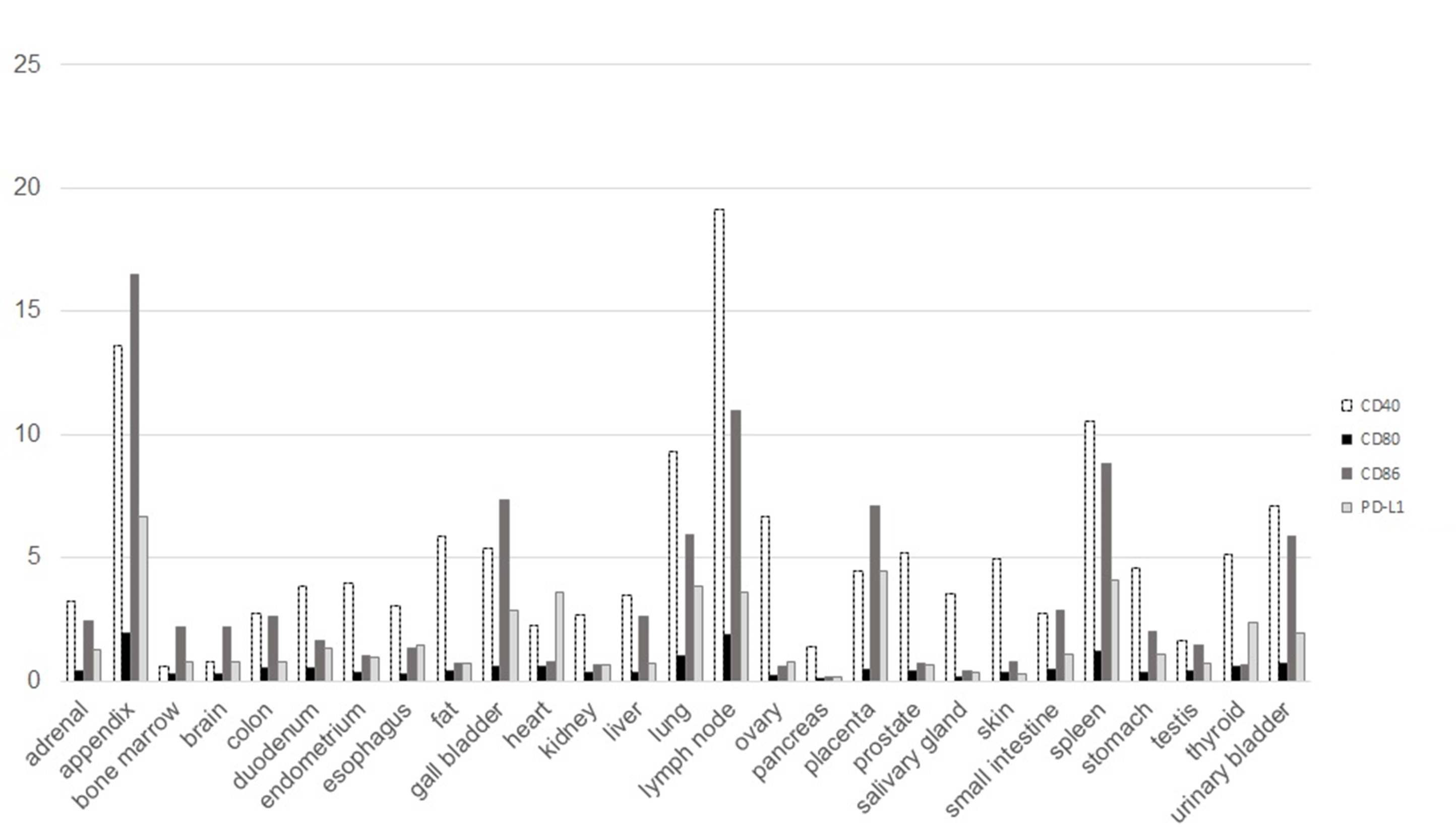

Supplement: Supplementary file 1 — Figure S1: Mean mRNA expression for 21 target genes in 27 different tissues (N = 95) analysed in the ‘HPA RNA‐seq normal tissues’ project (values are expressed as reads per kilobase per million reads placed, RPKM). S1A: HLA‐H, ‐G and MICA. S1B: HLA‐F, ‐F‐AS1 and MICB. S1C HLA‐E, ‐A, ‐B, and ‐C. S1D: HLA‐DRB1, ‐DQB1, and ‐DPB1. S1E: CD40, CD80, CD86 and PD‐L1. S1F: NLCR5, CIITA, INFg and TNF. [file TAN-106-e70390-s002.zip › tan70390-sup-0006-FigureS1@SupFig1E.jpg]

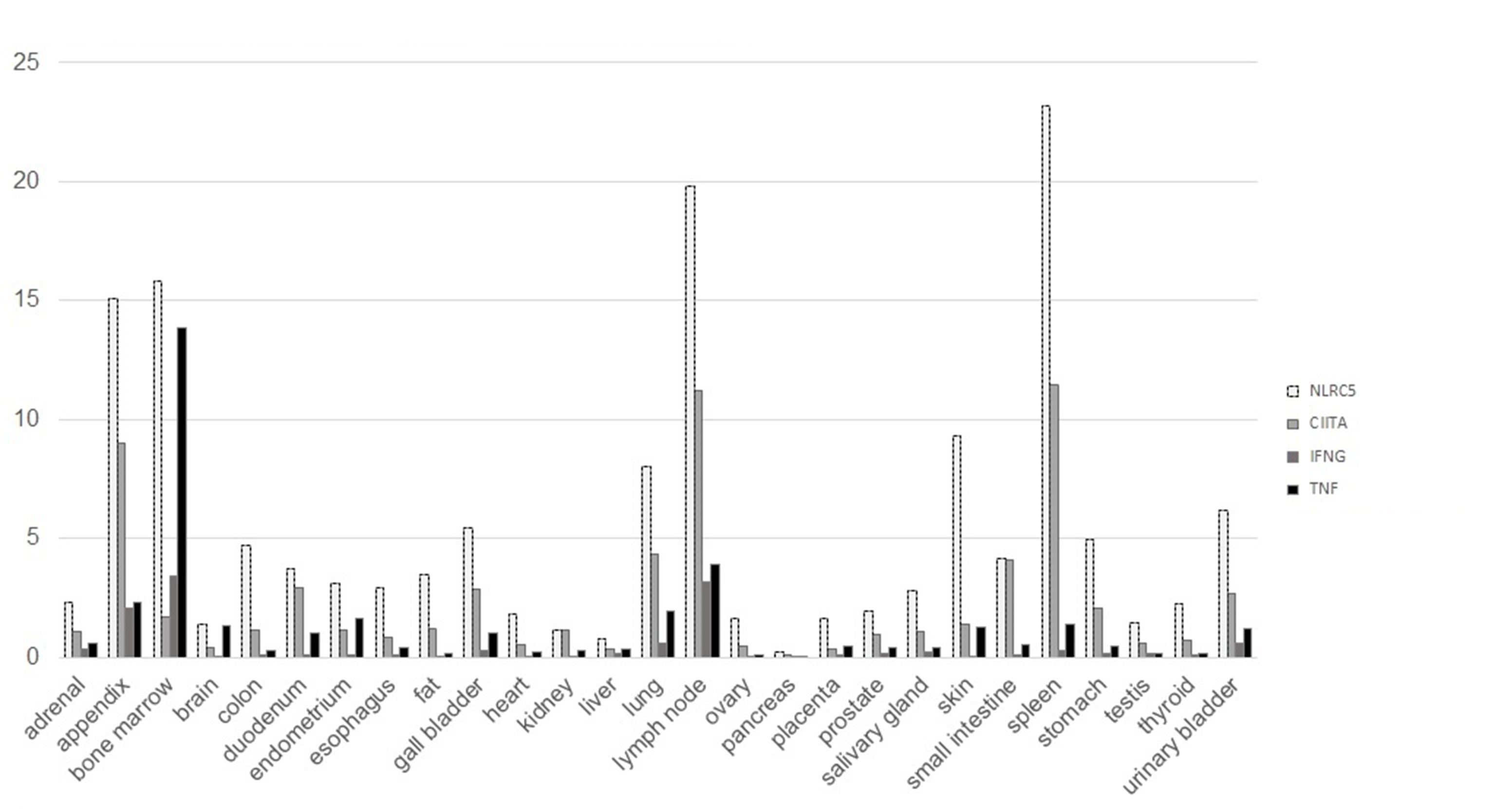

Supplement: Supplementary file 1 — Figure S1: Mean mRNA expression for 21 target genes in 27 different tissues (N = 95) analysed in the ‘HPA RNA‐seq normal tissues’ project (values are expressed as reads per kilobase per million reads placed, RPKM). S1A: HLA‐H, ‐G and MICA. S1B: HLA‐F, ‐F‐AS1 and MICB. S1C HLA‐E, ‐A, ‐B, and ‐C. S1D: HLA‐DRB1, ‐DQB1, and ‐DPB1. S1E: CD40, CD80, CD86 and PD‐L1. S1F: NLCR5, CIITA, INFg and TNF. [file TAN-106-e70390-s002.zip › tan70390-sup-0007-FigureS1@SupFig1F.jpg]

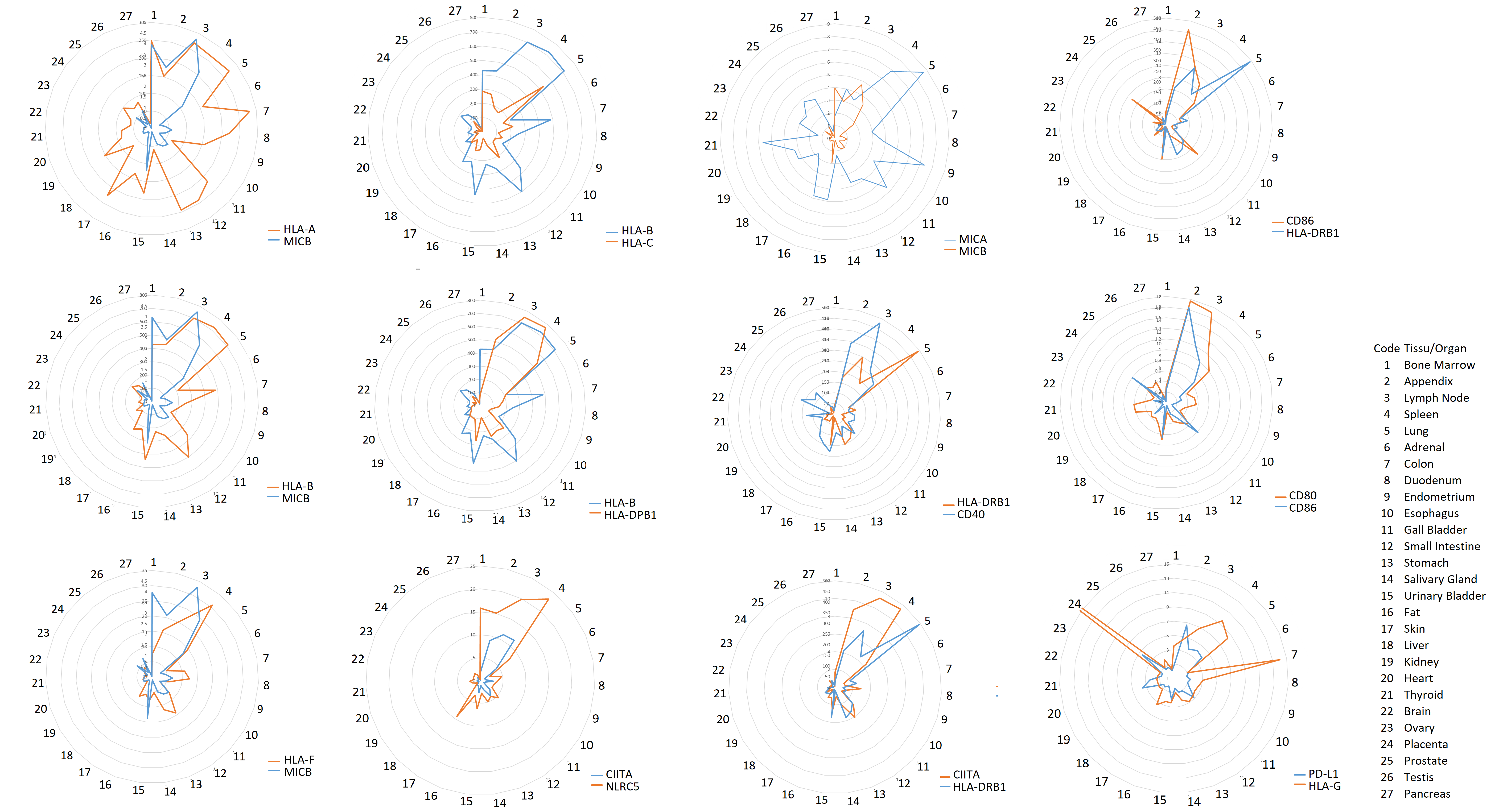

Supplement: Supplementary file 2 — Figure S2: Highlights of tissue‐specific gene expression that illustrates asymmetric correlation revealed in Figure 2. [file TAN-106-e70390-s004.tif]

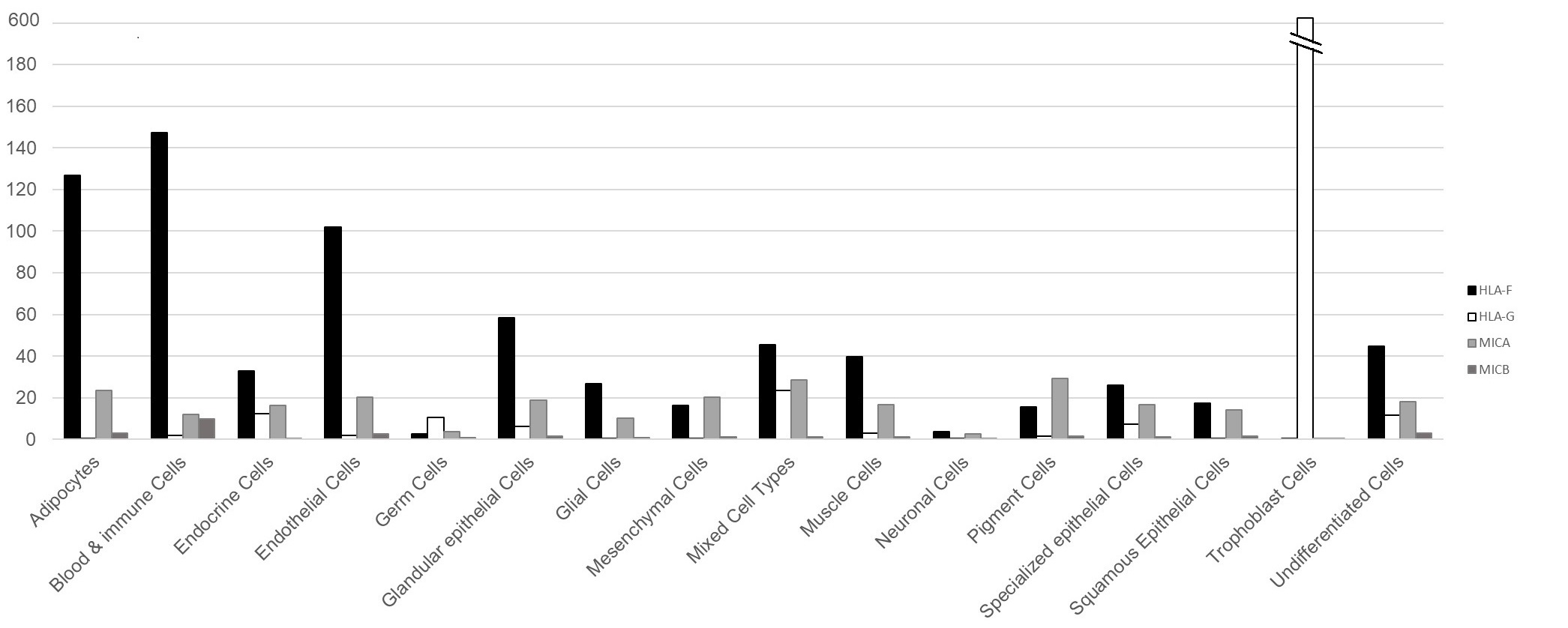

Supplement: Supplementary file 3 — Figure S3: Single‐cell RNA sequencing (scRNA‐seq) data for 18 of the 21 target genes in 31 healthy human tissues. Transcriptional expression is normalised to transcripts per million protein coding genes and expressed as ‘nTPM’. S3A: HLA‐F, ‐G, MICA and MICB. S3B: HLA‐E, ‐A, ‐B and ‐C. S3C: HLA‐DRB1, ‐DQB1 and ‐DPB1. S3D: CD40, CD80 and CD86. S3E: NLCR5, CIITA, INFg and TNF. [file TAN-106-e70390-s003.zip › tan70390-sup-0009-FigureS3@SupFig3A.jpg]

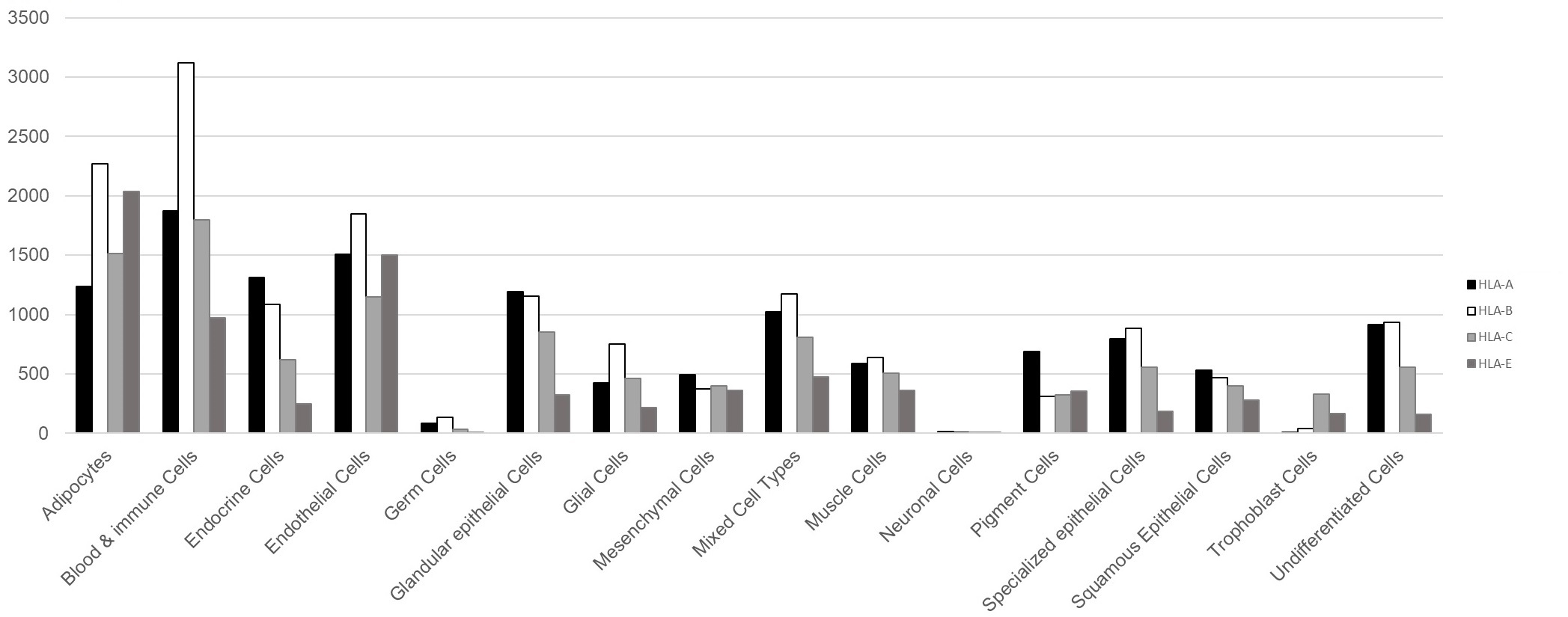

Supplement: Supplementary file 3 — Figure S3: Single‐cell RNA sequencing (scRNA‐seq) data for 18 of the 21 target genes in 31 healthy human tissues. Transcriptional expression is normalised to transcripts per million protein coding genes and expressed as ‘nTPM’. S3A: HLA‐F, ‐G, MICA and MICB. S3B: HLA‐E, ‐A, ‐B and ‐C. S3C: HLA‐DRB1, ‐DQB1 and ‐DPB1. S3D: CD40, CD80 and CD86. S3E: NLCR5, CIITA, INFg and TNF. [file TAN-106-e70390-s003.zip › tan70390-sup-0010-FigureS3@SupFig3B.jpg]

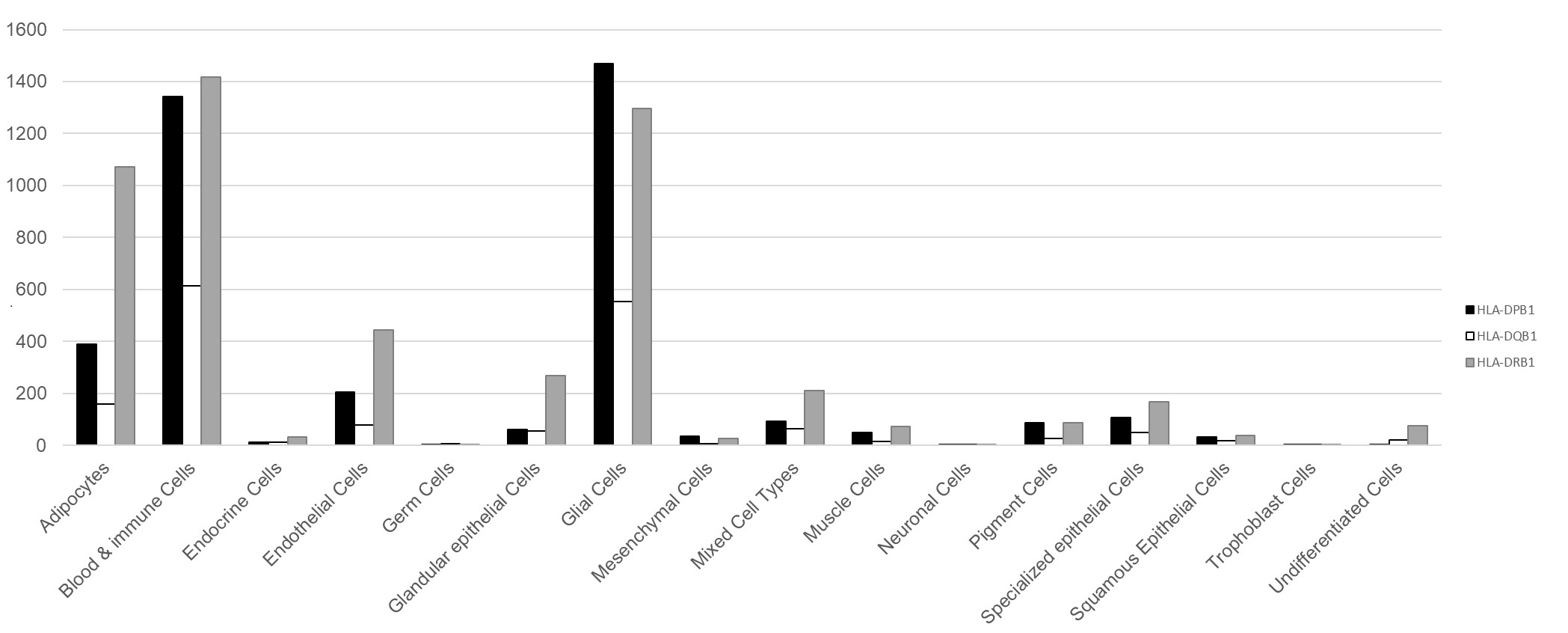

Supplement: Supplementary file 3 — Figure S3: Single‐cell RNA sequencing (scRNA‐seq) data for 18 of the 21 target genes in 31 healthy human tissues. Transcriptional expression is normalised to transcripts per million protein coding genes and expressed as ‘nTPM’. S3A: HLA‐F, ‐G, MICA and MICB. S3B: HLA‐E, ‐A, ‐B and ‐C. S3C: HLA‐DRB1, ‐DQB1 and ‐DPB1. S3D: CD40, CD80 and CD86. S3E: NLCR5, CIITA, INFg and TNF. [file TAN-106-e70390-s003.zip › tan70390-sup-0011-FigureS3@SupFig3C.jpg]

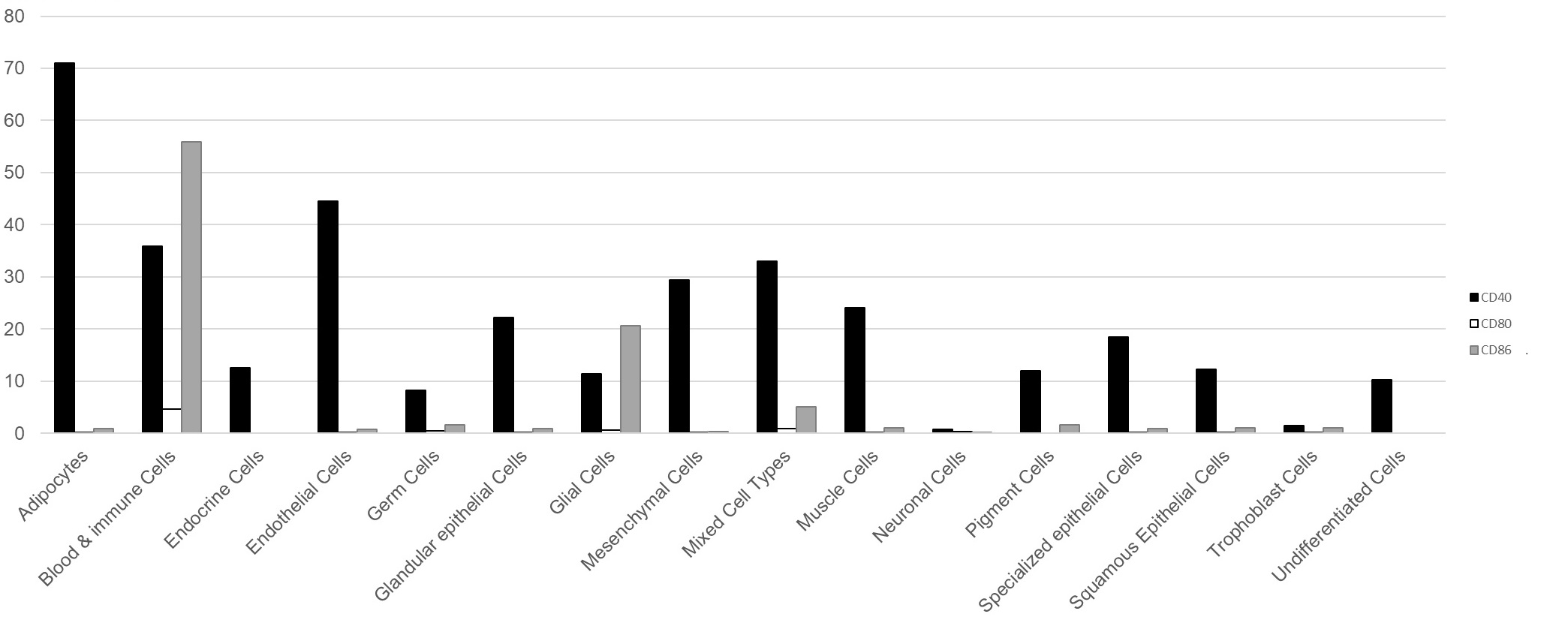

Supplement: Supplementary file 3 — Figure S3: Single‐cell RNA sequencing (scRNA‐seq) data for 18 of the 21 target genes in 31 healthy human tissues. Transcriptional expression is normalised to transcripts per million protein coding genes and expressed as ‘nTPM’. S3A: HLA‐F, ‐G, MICA and MICB. S3B: HLA‐E, ‐A, ‐B and ‐C. S3C: HLA‐DRB1, ‐DQB1 and ‐DPB1. S3D: CD40, CD80 and CD86. S3E: NLCR5, CIITA, INFg and TNF. [file TAN-106-e70390-s003.zip › tan70390-sup-0012-FigureS3@SupFig3D.jpg]

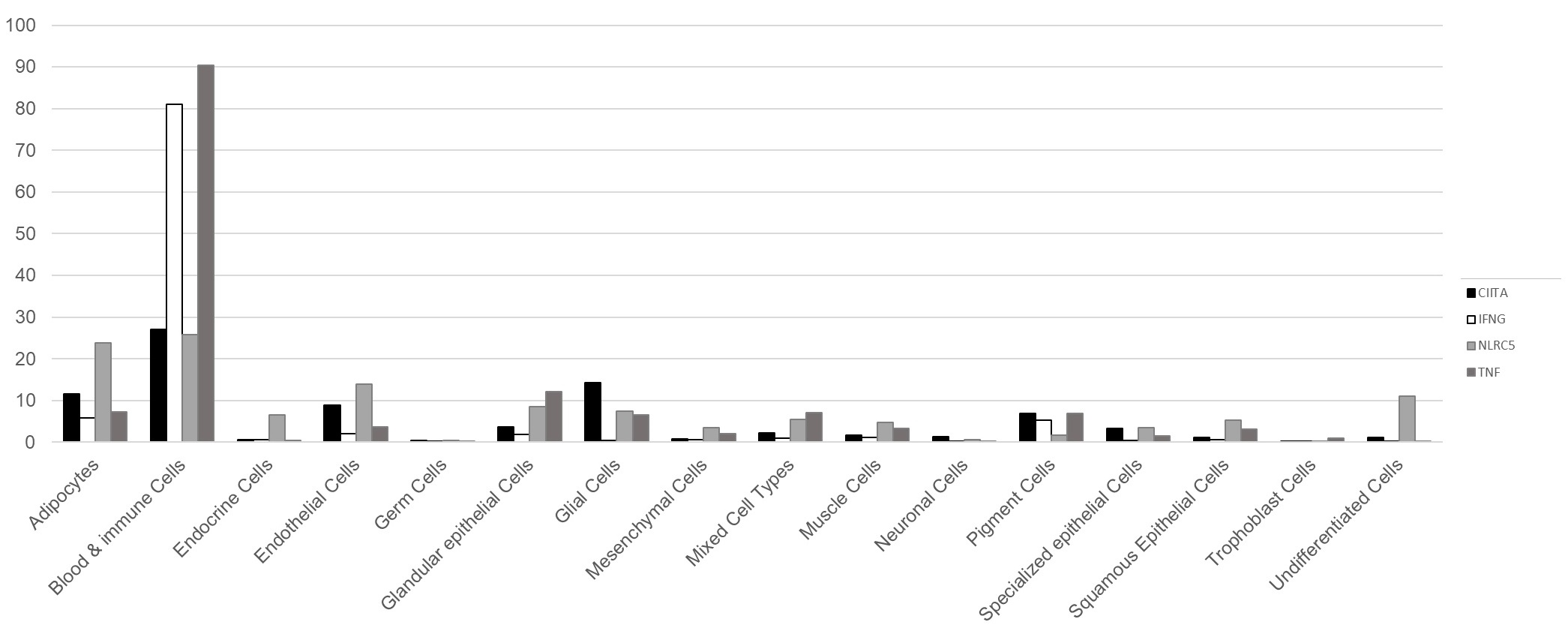

Supplement: Supplementary file 3 — Figure S3: Single‐cell RNA sequencing (scRNA‐seq) data for 18 of the 21 target genes in 31 healthy human tissues. Transcriptional expression is normalised to transcripts per million protein coding genes and expressed as ‘nTPM’. S3A: HLA‐F, ‐G, MICA and MICB. S3B: HLA‐E, ‐A, ‐B and ‐C. S3C: HLA‐DRB1, ‐DQB1 and ‐DPB1. S3D: CD40, CD80 and CD86. S3E: NLCR5, CIITA, INFg and TNF. [file TAN-106-e70390-s003.zip › tan70390-sup-0013-FigureS3@SupFig3E.jpg]
